# MABSA: A curated Malayalam aspect based sentiment analysis dataset on movie reviews

**DOI:** 10.1016/j.dib.2023.109452

**Published:** 2023-07-26

**Authors:** Syam Mohan E, R. Sunitha

**Affiliations:** Department of Computer Science, School of Engineering and Technology, Pondicherry University, Puducherry, India

**Keywords:** Malayalam sentiment analysis, Aspect, Movie review, Malayalam dataset

## Abstract

Regional languages are being used more frequently in online platforms as a result of the expanding use of digital technology. Understanding user opinions on social media platforms, forums, blogs, and other digital platforms that employ Indian regional languages has become significant due to their role in various applications. Research on sentiment analysis of Indian regional language texts suffers due to the unavailability of available regional language datasets. The curated Malayalam Aspect Based Sentiment Analysis (MABSA) dataset is a labeled dataset for Aspect Based Sentiment Analysis (ABSA) on the Indian regional language Malayalam over the movie review domain. Malayalam movie reviews, an excellent source of text data for ABSA, are collected from an online survey using Google form and manually collecting reviews from three social media platforms: IMDb, Facebook, and YouTube. Nine target aspects were identified, and three annotators annotated the dataset based on the sentiment polarity of each aspect. A total of 4000 reviews were collected, and a total of 7507 aspects are identified in the reviews. Spearman's correlation and Fleiss Kappa Index are used to analyze the annotated dataset's correlation. It has been found that the high correlation between the annotators implies that the MABSA dataset is of gold standard.

**Specifications Table**

**Table:** 

Subject	Computer Science, Natural Language Processing
Specific subject area	Sentiment Analysis, Aspect Based Sentiment Analysis, Text Classification, Machine Learning, Deep Learning
Type of data	Text Review
How the data were acquired	The Malayalam aspect-based sentiment analysis dataset was curated by two methods: using Google survey tools (Google Forms) and manually collecting movie reviews in Malayalam from three social media platforms: Facebook, YouTube, and IMDb.
Data format	Raw Filtered
Description of data collection	Movie reviews in Malayalam are collected through two ways. The former through a Google form, asking people to comment reviews about 20 movies. The latter through various groups and pages on social media platforms, viz. Facebook, YouTube, and IMDb websites. Reviews with at least one target aspect present were included in the dataset. The target aspects include direction, acting, songs, screenplay, story, casting, editing, cinematography, and BGM (background music). Three proficient annotators manually labeled the dataset according to the sentiment of each aspect present in every review. A copy of the Google form is provided as a supplementary document.
Data source location	Movie reviews in Malayalam are collected from https://www.facebook.com, https://www.youtube.com, and https://www.imdb.com.
Data accessibility	Repository name: Mendeley Data repository Data identification number: 10.17632/f3ftpd7xpg.3. Direct URL to data: https://data.mendeley.com/datasets/f3ftpd7xpg/3.

## Value of the Data

• MABSA dataset contains 4000 movie reviews in Malayalam and a total of 7507 aspects which are manually labeled based on sentiment polarity. To our knowledge, this will be the first publicly available dataset on Malayalam sentiment analysis.
  Based on Malayalam movie reviews, researchers can create new algorithms and models for sentiment analysis in the entertainment sector using the MABSA dataset. This may result in fresh perspectives and advancements in exploring natural language processing in Malayalam.
• Malayalam is a low-resource Indian regional language [1]. Hence this dataset will benefit the regional languages in modeling/proposing machine languages and deep language learning models for different applications like recommender systems, next-word prediction, etc.
• Malayalam is a morphologically rich Indian language that 38 million people worldwide speak [2]. The dataset on ABSA can provide light on local sentiment by analyzing sentiment conveyed in Malayalam text, which may differ from sentiment conveyed in other languages like English.

## 1 Objective

The primary objective of creating the MABSA dataset is to develop a high-quality, labeled dataset that properly depicts the attitudes expressed towards particular aspects or features of movies described in the Malayalam language. Since Malayalam is a low-resource language, the contribution of the MABSA dataset will be a huge asset to the research community.

## 2 Data Description

ABSA often relies on movie reviews as a source of data since they tend to offer detailed reviews and comments on diverse features or aspects of the film. MABSA dataset for movie reviews aims to pinpoint the individual aspects of the film that are expressed within the review comment in the Malayalam language and evaluates the corresponding sentiment orientation of every aspect present in the text. Sentiment orientations are mapped to positive, negative, or neutral depending on the words or phrases that describe each aspect. The description of each column in the MABSA dataset is presented in Table 1.

**Table 1:** Column description.

Column	Description
Id	This column contains individual reference numbers for each movie review.
Review	This column contains reviews of movies in Malayalam.
English Translation (Approximate)	This column contains the approximate translation of the reviews in the English language.
Direction	This column contains the sentiment label for the ‘direction' aspect of the movie.
Songs	This column contains the sentiment label for the ‘songs’ aspect of the movie.
Acting	This column contains the sentiment label for the ‘acting' aspect of the movie.
Screenplay	This column contains the sentiment label for the ‘screenplay’ aspect of the movie.
Story	This column contains the sentiment label for the ‘story' aspect of the movie.
Casting	This column contains the sentiment label for the ‘casting' aspect of the movie.
Editing	This column contains the sentiment label for the ‘editing' aspect of the movie.
Cinematography	This column contains the sentiment label for the ‘cinematography' aspect of the movie.
BGM	This column contains the sentiment label for the ‘background music’ aspect of the movie.

## 3 Experimental Design, Materials and Methods

**Fig. 1:**
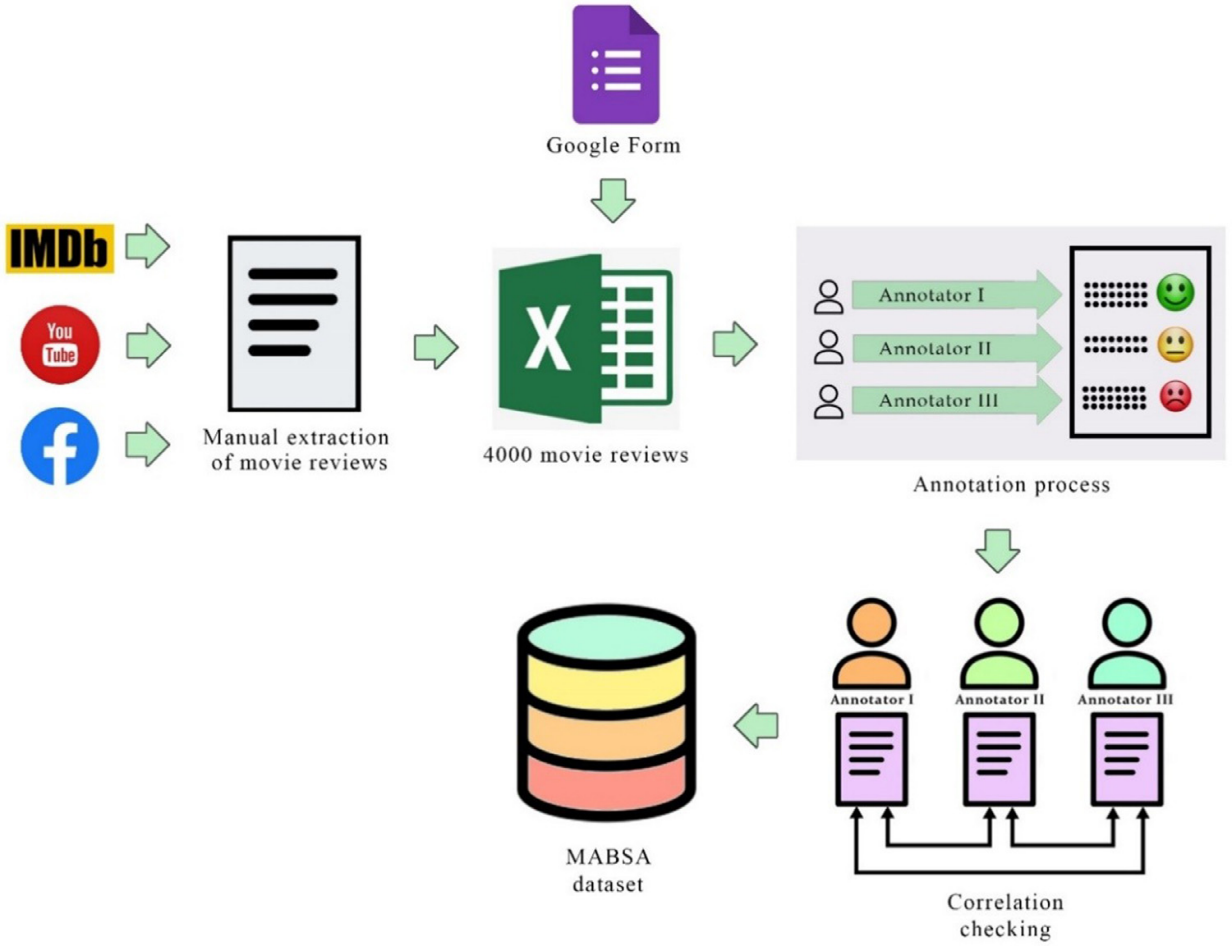
Work flow process for MABSA dataset creation.

The workflow process for MABSA dataset creation is depicted in Fig. 1. Movie reviews in Malayalam are collected through two ways. The former is through a Google form, asking people to comment reviews about 20 movies. The latter is from various groups and pages on social media platforms, viz. Facebook, YouTube, and IMDb websites. Reviews were collected based on the presence of at least one target aspect in the review, where the target aspects include direction, songs, acting, screenplay, story, casting, editing, cinematography, and background music. These nine target aspects are short-listed from a set of aspects related to the film industry, which people predominantly use when writing a review comment. A total of 4000 reviews of various movies are collected and stored in an Excel sheet.

**Fig. 2:**
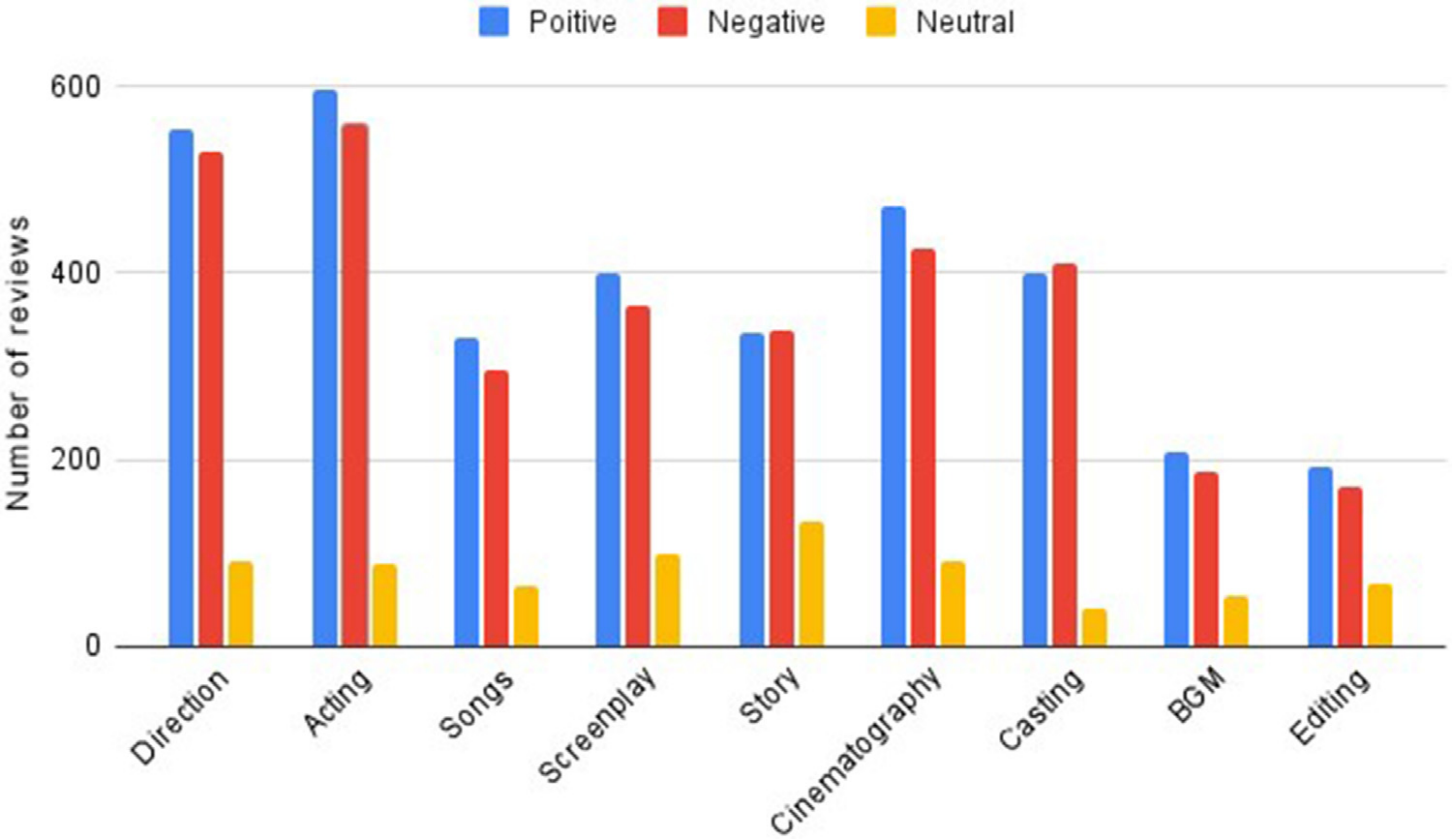
Distribution of aspects across sentiment classes.

In the next stage, these reviews are given to three proficient annotators for labeling. No specific protocol was followed to carry out the task of annotation. The top-ranked aspects of movie reviews are identified as the target aspects and given for annotation. The three annotators are native Malayalam speakers who are capable of identifying the aspects and any other term synonymous to the targeted aspects. Based on the nature of the comment on the target aspects present in a review, the annotators quantify the sentiment of each aspect as 1, 0, and 0.5 for positive, negative, and neutral, respectively. Table 2 shows an example of a movie review in Malayalam (with English translation). After annotating the entire collection of reviews, 7507 aspects are identified totally in the dataset. Table 3 presents the distribution of aspects across different sentiment classes which is illustrated in Fig. 2.

**Table 2:** Example for Malayalam movie review, the aspects present and their label values.

**Table 3:** Distribution of aspects to across sentiment classes.

	Sentiment		
Aspect	Positive	Negative	Neutral
Direction	554	531	90
Acting	596	560	89
Songs	332	297	64
Screenplay	401	365	99
Story	335	338	133
Cinematography	471	427	90
Casting	401	411	41
BGM	208	188	55
Editing	192	171	68

As a final step, the correlation between the three annotators is calculated using Spearman's correlation and Fleiss Kappa Index. These two methods will help to understand how well the agreement between the three annotators have been in labeling the dataset. Eq. (1) have been used to calculate Spearman's correlation [3].

(1) $$\rho =1-\;\frac{6\;\sum d_{i}^{2}}{n\left(n^{2}\;-1\right)}$$

Where *ρ* is the Spearman's correlation coefficient, d is the difference between the two annotator's agreement on aspect sentiment and n is the number of annotations. The annotation correlation was also evaluated using the Fleiss Kappa Index using Eq. (2) [4].

(2) $$\kappa =\;\frac{\bar{P}-\;\bar{P_{e}}}{1-\;\bar{P_{e}}}$$

Where κ is the Fleiss Kappa value, $\bar{P}$ is the observed proportion between a pair of annotators agreement and $\bar{P_{e}}$ is the expected proportion between the annotator agreement. Table 3 summarizes the results of the two correlation analyses. The results of the correlation analysis on annotator agreements exhibit a high correlation between the three annotators. Hence the dataset created is a gold standard for Malayalam ABSA on movie reviews. The annotator 1 dataset, one among the high correlations exhibiting datasets between the annotator combinations, is chosen as the final dataset for MABSA. Now this labeled dataset in Excel format is converted to CSV format for easy usage of the dataset (Table 4).

**Table 4:** Correlation between annotators.

Annotator combinations	Spearman's correlation	Fleiss Kappa Index
Annotator I & Annotator II	0.8881673	0.896
Annotator I & Annotator III	0.8013735	0.815
Annotator II & Annotator III	0.8207227	0.828

## Ethics Statements

Data collected was completely anonymous, and the data redistribution policies of social media platforms have been complied with.

## CRediT authorship contribution statement

**Syam Mohan E:** Conceptualization, Data curation, Writing – original draft. **R. Sunitha:** Supervision, Writing – review & editing.

## Data Availability

MABSA Dataset (Original data) (Mendeley Data).

## Appendix Supplementary materials

Image, application 1
