# Supplementary material for: MABSA: A curated Malayalam aspect based sentiment analysis dataset on movie reviews
Source: Data Brief. 2023 Jul 26;50:109452. doi: 10.1016/j.dib.2023.109452 (PMC10415832; doi:10.1016/j.dib.2023.109452)
Supplement: Supplementary file 1 [file mmc1.pdf]

Malayalam Movie Review Data Collection

നമസ്കാരം,
മലയാള സിനിമ നിരൂപണങ്ങളുടെ കൃത്യമായ മലയാളം ഡാറ്റാസെറ്റ് (dataset) നിലവിൽ ഇല്ലാത്തതിനാൽ, എന്റെ ഗവേഷണത്തിന്റെ ഭാഗമായി സ്വകീയമായ ഒരു മലയാളം ഡാറ്റാസെറ്റ് ഉണ്ടാക്കുന്നതിനു വേണ്ടിയാണ് ഈ സർവ്വേ. നിങ്ങൾക്ക് സുപരിചിതമായ കുറച്ച് സിനിമകളുടെ പേരുകൾ താഴെ കൊടുത്തിരിക്കുന്നു. ആ സിനിമകളെ കുറിച്ചുള്ള നിങ്ങളുടെ അഭിപ്രായം 3 വരികളിൽ കുറയാതെ മലയാളം ഭാഷയിൽ (ഇംഗ്ലീഷ് & മംഗ്ലീഷ് പാടില്ല) എഴുതുവാൻ താല്പര്യപ്പെടുന്നു. Review എഴുതുമ്പോൾ താഴെ കൊടുത്തിരിക്കുന്ന വിഷയങ്ങൾ ചർച്ച ചെയ്യും വിധം നിരൂപണമെഴുതുവാൻ ശ്രദ്ധിക്കുക.
- സംവിധാനം -അഭിനയം - സംഗീതം - തിരക്കഥ -കഥ - ഛായാഗ്രഹണം -കഥാപാത്രങ്ങളുടെ തിരഞ്ഞെടുപ്പ്(casting) -പശ്ചാത്തല സംഗീതം - എഡിറ്റിംഗ്

നിങ്ങൾ കാണാത്ത സിനിമ ആണ് എങ്കിൽ 'NIL' എന്ന് എഴുതുക.

Greetings,
Since there is currently no accurate Malayalam dataset of Malayalam movie reviews, this survey is to create a personal Malayalam dataset as part of my research. Below are the names of few movies that you may be familiar with. Feel free to write your opinion about those movies in Malayalam language (not English & Manglish) in not less than 3 lines. While writing the review, make sure to write the review in such a way that the following topics are discussed.
- Direction - Acting - Music - Screenplay - Story - Cinematography - Casting - Background Music - Editing

If it is a movie you have not seen then write 'NIL'.

Syam Mohan E
PhD Scholar
Department of Computer Science
Pondicherry University
syammohane@gmail.com

* Indicates required question

1. Name *

2. Age *

3. Gender *

Mark only one oval.

- Male
- Female
- Other

4. Qualification *

Mark only one oval.

- Post graduate
- Graduate
- Matriculation

5. 1. ദൃശ്യം | Drishyam *

6. ദൃശ്യം | Drishyam overall opinion *

Mark only one oval.

- Good
- Bad
- Neutral

7. 2. അഞ്ചാം പാതിര | Ancham Pathira *

8. അഞ്ചാം പാതിര | Ancham Pathira overall opinion *

Mark only one oval.

- Good
- Bad
- Neutral

9. 3. ചുരുളി | Churuli *

10. ചുരുളി | Churuli overall opinion *

Mark only one oval.

- Good
- Bad
- Neutral

11. 4. ഹൃദയം | Hridhayam *

12. ഹൃദയം | Hridhayam overall opinion *

Mark only one oval.

- Good
- Bad
- Neutral

13. 5. കുമ്പളങ്ങി നൈറ്റ്സ് | Kumbalangi Nights *

14. കുമ്പളങ്ങി നൈറ്റ്സ് | Kumbalangi Nights overall opinion *

Mark only one oval.

- Good
- Bad
- Neutral

15. 6. ജെല്ലിക്കെട്ട് | Jellikettu *

____

____

____

____

____

16. ജെല്ലിക്കെട്ട് | Jellikettu overall opinion *

Mark only one oval.

- Good
- Bad
- Neutral

17. 7. ജോസഫ് | Joseph *

____

____

____

____

____

18. ജോസഫ് | Joseph overall opinion *

Mark only one oval.

- Good
- Bad
- Neutral

19. 8. ഒടിയൻ | Odiyan *

20. ഒടിയൻ | Odiyan overall opinion *

Mark only one oval.

- Good
- Bad
- Neutral

21. 9. ആറാട്ട് | Arattu *

22. ആറാട്ട് | Arattu overall opinion *

Mark only one oval.

- Good
- Bad
- Neutral

23. 10. സലാല മൊബൈൽസ് | Sala Mobiles *

24. സലാല മൊബൈൽസ് | Sala Mobiles overall opinion *

Mark only one oval.

- Good
- Bad
- Neutral

25. 11. മായാമോഹിനി | Mayamohini *

26. മായാമോഹിനി | Mayamohini overall opinion *

Mark only one oval.

- Good
- Bad
- Neutral

27. 12. ഭാസ്കർ ദി റാസ്കൽ | Baskar the raskal *

28. ഭാസ്കർ ദി റാസ്കൽ | Baskar the raskal overall opinion *

Mark only one oval.

- Good
- Bad
- Neutral

29. 13. വരനെ ആവശ്യമുണ്ട് | Varane Avashyamund *

30. വരനെ ആവശ്യമുണ്ട് | Varane Avashyamund overall opinion *

Mark only one oval.

- Good
- Bad
- Neutral

31. 14. ലവ് ആക്ഷൻ ഡ്രാമ | Love Action Drama *

32. ലവ് ആക്ഷൻ ഡ്രാമ | Love Action Drama overall opinion *

Mark only one oval.

- Good
- Bad
- Neutral

33. 15. ഇവൻ മര്യാദരാമൻ | Ivan Maryadharaman *

34. ഇവൻ മര്യാദരാമൻ | Ivan Maryadharaman overall opinion *

Mark only one oval.

- Good
- Bad
- Neutral

35. 16. കമ്മത്ത് ആൻഡ് കമ്മത്ത് | Kammath and Kammath *

36. കമ്മത്ത് ആൻഡ് കമ്മത്ത് | Kammath and Kammath overall opinion

Mark only one oval.

- Good
- Bad
- Neutral

37. 17. കാസനോവ | Casanova *

38. കാസനോവ | Casanova overall opinion *

Mark only one oval.

- Good
- Bad
- Neutral

39. 18. പെരുച്ചാഴി | Peruchazhi *

40. പെരുച്ചാഴി | Peruchazhi overall opinion *

Mark only one oval.

- Good
- Bad
- Neutral

41. 19. സൗണ്ട് തോമ | Sound Thoma *

42. സൗണ്ട് തോമ | Sound Thoma overall opinion *

Mark only one oval.

- Good
- Bad
- Neutral

43. 20. ദി ഗ്രേറ്റ് ഇന്ത്യൻ കിച്ചൻ | The Great Indian Kitchen *

44. ദി ഗ്രേറ്റ് ഇന്ത്യൻ കിച്ചൻ | The Great Indian Kitchen overall opinion *

Mark only one oval.

- Good
- Bad
- Neutral

This content is neither created nor endorsed by Google.

Google Forms
